# A 50-year-old refugee woman with a lithopedion and a lifetime of trauma: a case report

**DOI:** 10.1186/s12905-023-02244-z

**Published:** 2023-03-07

**Authors:** Waseem Sous, Michaela Sous, Ayorinde Soipe, Amy E. Caruso Brown, Andrea V. Shaw

**Affiliations:** 1 Department of Internal Medicine, SUNY Upstate Medical University, 750 East Adams St, Syracuse, NY 13210 USA; 2 Division of Hospital Medicine, University of California, San Francisco, San Francisco, CA USA; 3 HEAL Initiative, University of California, San Francisco, San Francisco, CA USA; 4 Department of Obstetrics & Gynecology, SUNY Upstate Medical University, Syracuse, NY USA; 5 Center for Bioethics and Humanities, SUNY Upstate Medical University, Syracuse, NY USA; 6 Department of Pediatrics, SUNY Upstate Medical University, Syracuse, NY USA; 7 Institute for Global Health and Translational Science, SUNY Upstate Medical University, Syracuse, NY USA

**Keywords:** Lithopedion, Fetal demise, Medical distrust, Refugee

## Abstract

**Background:**

Lithopedion is a term that refers to a fetus that has calcified or changed to bone. The calcification may involve the fetus, membranes, placenta, or any combination of these structures. It is an extremely rare complication of pregnancy and can remain asymptomatic or present with gastrointestinal and/or genitourinary symptoms.

**Case presentation:**

A 50-year-old Congolese refugee with a nine-year history of retained fetus after a fetal demise was resettled to the United States (U.S.). She had chronic symptoms of abdominal pain and discomfort, dyspepsia, and gurgling sensation after eating. She experienced stigmatization from healthcare professionals in Tanzania at the time of the fetal demise and subsequently avoided healthcare interaction whenever possible. Upon arrival to the U.S., evaluation of her abdominal mass included abdominopelvic imaging which confirmed the diagnosis of lithopedion. She was referred to gynecologic oncology for surgical consultation given intermittent bowel obstruction from underlying abdominal mass. However, she declined intervention due to fear of surgery and elected for symptom monitoring. Unfortunately, she passed away due to severe malnutrition in the context of recurrent bowel obstruction due to the lithopedion and continued fear of seeking medical care.

**Conclusion:**

This case demonstrated a rare medical phenomenon and the impact of medical distrust, poor health awareness, and limited access to healthcare among populations most likely to be affected by a lithopedion. This case highlighted the need for a community care model to bridge the gap between the healthcare team and newly resettled refugees.

## Background

The word *lithopedion* is derived from the Greek *lithos*, meaning stone, and *paidion*, meaning child [1]. Lithokelyphos (stone sheath or egg shell) refers to calcification of the membranes alone. Lithokelyphepedion (stone sheath child) refers to calcification of the membranes and the fetus. Lithopedion (stone child) refers to calcification of the fetus with negligeable calcification of the membranes [1]. Lithopedion was first described in the tenth century by Albucasis, a surgeon of the Arabic era of medicine [2].

The occurrence of a lithopedion is extremely rare. Fewer than 300 cases have been reported since the discovery of a case in France in 1582 [3–6]. It occurs in an estimated 1.5 to 1.8% of extrauterine pregnancies and in 0.00045% of all pregnancies [7]. Cases have been reported in women between the ages of 23 to 100 years with an estimated interval of retention of 4 to 60 years [3]. When fetal demise ensues and the fetus is too large to be reabsorbed, the immune system considers it as a foreign body and induces calcium-rich substance deposition thereby turning the fetus into stone [7].

Here we report a case of a refugee woman with a lithopedion that occurred 9 years prior to resettlement, but remained a physical and psychological burden she bore due to a global distrust of healthcare.

## Case presentation

A 50-year-old postmenopausal G9P8105 Congolese refugee presented to our health system within 30 days of arrival to the United States (U.S.) with symptoms of abdominal pain and discomfort, chronic dyspepsia, and gurgling sensation after eating for many years. During her initial office visit, review of records from the mandatory health clearance evaluation completed 6 months prior to arrival in the U.S. listed her medical problems as “Calcified Abdominal Pregnancy and Essential Hypertension.” Fetal biometry estimated 28 weeks gestational age at time of fetal demise. She provided a history of 8 spontaneous vaginal deliveries with 3 children who died shortly after birth. She separated from her husband and had her 9^th^ pregnancy from another relationship. Occurring 9 years prior to resettlement, this final pregnancy had a preterm fetal demise without passage of products of conception, resulting in a large calficied abdominal mass. The patient’s only report of medical history involved high blood pressure for which an antihypertensive was prescribed, although she did not take the medication as she felt “fine.” She had no surgical history. She had no self-reported personal or family history of chronic kidney disease (CKD). She denied taking any over-the-counter medications, herbals, or supplements. On review of symptoms, she had no genitourinary (GU) or constitutional complaints. Her last menstrual cycle was 2 years prior. Her life story involved a traumatic past as she was born into a Congolese family amidst the years of fleeing war for Burunndi. Subsequent violence in Burundi forced her family to flee to a refugee camp in Tanzania, where she started a family of her own and stayed until her resettlement in the U.S. Her literacy level was low as she completed primary school in Burundi and had partial literacy in Swahili.

Her initial office visit physical examination revealed a blood pressure of 162/83 and a firm mobile lower abdominal mass, measuring approximately 15 cm by 20 cm, without tenderness to palpation. When she was asked further about her understanding of this condition, she recounted a difficult story. When she was pregnant with her 9^th^ child, she noticed decreased fetal movement. She presented to the health facility in the refugee camp and was informed about the lack of a fetal heartbeat indicating demise of the fetus. She was instructed to go home and try to “deliver” the fetus and to return in two weeks if nothing happened spontaneously. She returned to the health facility as instructed, but was accused of “evil works,” “taking drugs,” and “killing the baby.” The medical provider at the refugee health facility recommended interventions to remove the fetus, but she did not feel comfortable with this care plan after the verbal abuse she met upon arrival. She went home to pray and declined to return to the clinic until the mandatory health clearance 6 months prior to departure for resettlement in the U.S.

One week after her initial office visit in the U.S., she presented to the emergency department for evaluation of nausea and intractable vomiting. She was found to be hypokalemic with a potassium of 3 mmol/L. An abdominal x-ray showed a fetal skeleton and non-specific bowel gas pattern with mild distention of small bowel loops suggestive of an ileus. She was admitted overnight for clinical observation. Despite being accompanied by a Swahili-speaking health navigator, the patient was fearful of the hospital and declined all oral intake. She subsequently disclosed episodic symptoms of bowel obstruction in the refugee camp, and an episode of bilious emesis requiring several days of bowel rest. She avoided healthcare facilities during those times of sickness.

**Fig. 1:**
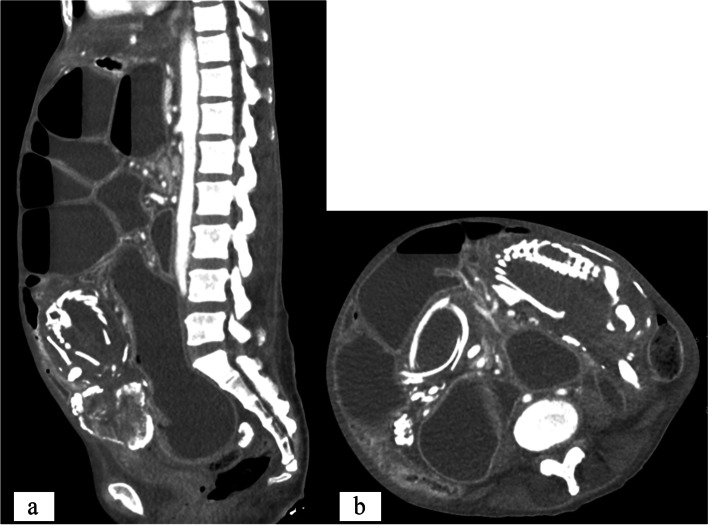
**a** Sagittal image showing fetal bony parts consistent with a lithopedion and dilated loops of small bowel, concerning for small bowel obstruction. **b** Axial image showing bony fetal structures and air-fluid level suggestive of bowel obstruction

Laboratory workup revealed a negative beta-human chorionic gonadotropin and CKD stage 2 (Table 1). Abdominopelvic computed tomography (CT) scan with intravenous and oral contrast showed findings consistent with a lithopedion within the anterior lower abdomen and anterior upper pelvis (Fig. 1a), possibly external to the uterus representing an ectopic pregnancy. CT also revealed significant compression of the pelvic structures and small bowel obstruction (Fig. 1b) in addition to significant compression of the inferior vena cava with venous collaterals within the body wall of the pelvic and hip regions.

**Table 1:** Initial laboratory data^a^

White-cell count	6.2
Hemoglobin	12.7
Platelets	231
Blood urea nitrogen	15
Creatinine	0.93
Glomerular filtration rate	71
Sodium	141
Potassium	4.0
Calcium	9.1
Albumin	3.9
Phosphorus	2.9
Alanine aminotransferase	14
Aspartate aminotransferase	22
Alkaline phosphatase	73
Total bilirubin	0.4

^a^Reference ranges are as follows: white-cell count, 4 × 10^3^ to 10 × 10^3^ per cubic millimeter; hemoglobin, 11.5 to 15.5 g per deciliter; platelets, 150 × 10^3^ to 400 × 10^3^ per cubic millimeter; blood urea nitrogen, 6 to 20 mg per deciliter; creatinine, 0.5 to 0.90 mg per deciliter; glomerular filtration rate > 60 ml per minute per 1.73 square meter; sodium, 136 to 145 mmol per liter; potassium 3.4 to 5.1 mmol per liter; calcium 8.6 to 10 mg per deciliter; albumin 3.5 to 5.2 g per deciliter; phosphorus 2.5 to 4.5 mg per deciliter; alanine aminotransferase, < 33 units per liter; aspartate aminotransferase < 32 units per liter; alkaline phosphatase 35 to 104 units per liter; total bilirubin < 1.2 mg per deciliter. To convert the values for blood urea nitrogen to millimoles per liter, multiply by 0.357. To convert the values for creatinine to micromoles per liter, multiply by 88.4

The patient was referred to nephrology given her hypertension and CKD stage 2. Urinalysis with microscopy was unremarkable and urine random total protein/creatinine ratio was 0.15 (ratio < 0.2 mg per milligram is considered within normal limits [8]). Workup for secondary causes of hypertension was largely unremarkable (Table 2). She was on a renin–angiotensin–aldosterone system (RAAS) blocking medication, lisinopril, during initial testing for secondary causes of hypertension. Repeat testing for primary hyperaldosteronism and a doppler ultrasound of the renal arteries were recommended but not completed. Previous laboratory data were unavailable to determine any temporal relationship between CKD and hypertension.

**Table 2:** Laboratory data for secondary causes of hypertension^a^

Thyroid stimulating hormone	2.840
Aldosterone	1.4
Renin activity	< 0.167
Aldosterone/renin ratio	> 8.4
Metanephrine Fractionated Plasma: Normetanephrine, Metanephrine	73.4, 45.6

^a^Reference ranges are as follows: thyroid stimulating hormone, 0.270 to 4.200 milli-international units per milliliter; aldosterone, 0.0 to 30.0 nanogram per deciliter; renin activity, 0.167 to 5.380 nanogram per milliliter per hour; aldosterone/renin ratio, 0.0 to 30 nanogram per deciliter per nanogram per milliliter per hour; normetanephrine, 0.0 to 218.9 picograms per milliliter; metanephrine, 0.0 to 88 picograms per milliliter

Her symptom of dyspepsia improved after *Helicobacter pylori* was diagnosed by stool antigen testing and treated with a 14-day course of oral Omeprazole 40 mg twice daily, Clarithromycin 500 mg twice daily, Amoxicillin 1 g twice daily, and Metronidazole 500 mg twice daily. Her blood pressure was controlled with oral Lisinopril 40 mg daily. After extensive counseling by specialists and primary care provider regarding surgical intervention, she declined, repeatedly stating “I just do not have it in my heart to do.” The patient’s perception of her health condition was directly related to a “spell” that someone in Tanzania had cast on her. She reassured the team that “I will let you know when I am ready; I am not scared of death.” She had recurrent symptoms of bowel obstruction requiring hospitalization and continued to decline medical and surgical care with the specialists and primary care physician after her discharge. Unfortunately, she passed away at home 14 months after resettlement from severe malnutrition in the context of recurrent bowel obstruction and continued fear of seeking medical care.

## Discussion and conclusions

This patient’s chronic intermittent abdominal pain and discomfort combined with findings of bowel obstruction and calcified fetal parts on imaging were suggestive of a symptomatic lithopedion. Other differential diagnosis of her gastrointestinal (GI) symptoms included *Helicobacter pylori *gastritis for which she received clarithromycin-based concomitant therapy given positive stool antigen testing, colorectal cancer, and inflammatory bowel disease. Differential diagnosis for a calcified abdominal mass included ovarian tumors, uterine fibroids, urinary tract neoplasms, or epiploic calcifications [9]. Though most medical texts reported that there are no classic clinical signs or symptoms [2, 9] to aid in the diagnosis of a lithopedion and the majority of cases are discovered during routine imaging or unrelated surgical procedures, the late diagnosis reflects a failure of history taking on the part of the healthcare professionals. In most reported cases, the affected women were aware that they had experienced a pregnancy that did not conclude with the birth of a child or even the passage of blood and tissue to suggest a miscarriage [1–7, 9–15]. The occurrence of the condition itself reflects lack of access to healthcare, due to multifactorial and intersecting social and structural factors, including poverty, geography, health literacy and awareness, distrust, and discrimination in healthcare. This patient experienced stigmatization from healthcare professionals at the time of the fetal demise that led her to avoid and distrust subsequent healthcare for her condition.

The clinical course of lithopedion is unpredictable. Most cases remain asymptomatic for extended periods of time or present with abdominal pain and GI or GU system obstruction [2, 9]. Some complications of a lithopedion include pelvic abscess and/or fistula formation, cephalopelvic disproportion in future pregnancies, extrusion of fetal parts through the abdominal wall, infertility, and lithopedion-induced malignancy [10–12].

Review of literature revealed cases of lithopedion resulting in mechanical bowel obstruction [13–15]. One study [14] showed a lithopedion in the right upper quadrant causing small bowel obstruction and subsequent bowel necrosis. Surgical intervention encountered bowel perforation and hemoperitoneum, and the patient passed away secondary to postoperative sepsis [14]. Another study reported intestinal obstruction and perforation of the cecum due to volvulus caused by adhesions of an ascending colon to a retrocolic lithopedion [15]. An additional study [3] demonstrated significant distortion of pelvic anatomy causing infertility. After surgical removal of the lithopedion and restoration of pelvic anatomy, fertility was regained [3]. Although review of the literature did not reveal any cases of lithopedion associated with CKD, a calcified abdominal mass can lead to distortion of pelvic anatomy [3] and compression of adjacent structures, including vasculature, as seen in this patient’s CT imaging. Thus, the nephrology team hypothesized that this patient’s pelvic mass caused chronic compression of the renal artery leading to RAAS activation, renovascular hypertension [16], and subsequent CKD.

This case of a rare medical phenomenon illustrates how medical distrust can persist across borders, beginning in Tanzania for our patient and extending to her experiences in healthcare settings in the U.S. Previous studies have shown that African immigrants may lack trust in the U.S. healthcare system and believe that some of their fellow immigrants experienced a deterioration in health status after receiving Western medical treatment [17]. In addition to medical distrust, individuals from countries with widespread witchcraft beliefs, such as Tanzania and the Democratic Republic of the Congo, show lower levels of life satisfaction, poor state of health, and higher degree of fatalism [18]. One study showed that witchcraft was a major deterrent to accessing HIV care after diagnosis and how it can be reinforced by family and friends [19]. However, this study also illustrated that the community healthcare workers’ knowledge and ability to impart medical education helped influence the acceptability of HIV care postdiagnosis [19].

Due to the rarity of this condition, poor health awareness, limited access to healthcare facilities [3], and medical distrust among populations most likely to be affected by lithopedion, physicians may have difficulty navigating and discussing this medical condition with patients. To bridge the gap between the healthcare team and this newly resettled refugee, a health navigator and care manager were closely involved in her case. Although she remained fearful of the recommended interventions, the health navigator and care manager played an integral role in providing interpretation assistance and support in navigating the healthcare system. Despite these efforts, the patient passed away due to severe malnutrition related to recurrent bowel obstruction and continued medical distrust stemming from her prior life experiences. This case demonstrated a rare medical phenomenon and the long-lasting and unfavorable impact of medical distrust, poor health awareness, and limited access to healthcare among populations most likely to be affected by a lithopedion. Healthcare professionals are encouraged not only to gain more knowledge about this rare condition, but also to explore underlying sociocultural and structural factors and to involve a community-engaged team early in the care to bridge the gap between the patient and the healthcare team.

## Data Availability

The data used during the current study are not publicly available to ensure that patient’s privacy is not compromised but are available from the corresponding author on reasonable request.

## Abbreviations

GI: Gastrointestinal
GU: Genitourinary
U.S.: United States
CKD: Chronic Kidney Disease
CT: Computed Tomography
RAAS: Renin–angiotensin–aldosterone system
